# Pelvic Floor Rehabilitation After Prostatectomy: Baseline Severity as a Predictor of Improvement—A Prospective Cohort Study

**DOI:** 10.3390/jcm14124180

**Published:** 2025-06-12

**Authors:** Małgorzata Terek-Derszniak, Małgorzata Biskup, Tomasz Skowronek, Mariusz Nowak, Justyna Falana, Jarosław Jaskulski, Mateusz Obarzanowski, Stanislaw Gozdz, Pawel Macek

**Affiliations:** 1 Holycross Cancer Centre, Department of Rehabilitation, Artwinskiego 3, 25-734 Kielce, Poland; malgorzatater@onkol.kielce.pl (M.T.-D.); malgorzatabi@onkol.kielce.pl (M.B.); tomaszsk@onkol.kielce.pl (T.S.); mariuszno@onkol.kielce.pl (M.N.); 2 Collegium Medicum, Jan Kochanowski University of Kielce, Zeromskiego 5, 25-369 Kielce, Poland; jaroslawja@onkol.kielce.pl (J.J.); mateuszob@onkol.kielce.pl (M.O.); stanislawgozdz1@gmail.com (S.G.); 3 Holycross Cancer Centre, Oncology Clinic, Artwinskiego 3, 25-734 Kielce, Poland; justynafa@onkol.kielce.pl; 4 Holycross Cancer Centre, Department of Urology, Artwinskiego 3, 25-734 Kielce, Poland; 5 Holycross Cancer Centre, Scientific Research, Epidemiology and R&D Centre, Artwinskiego 3, 25-734 Kielce, Poland

**Keywords:** prostate cancer, prostatectomy, incontinence, pelvic floor physical therapy

## Abstract

**Background/Objectives**: Urinary incontinence (UI) is a frequent and distressing complication after radical prostatectomy (RP). Pelvic floor muscle training (PFMT) is widely recommended as first-line rehabilitation, yet the clinical factors influencing its effectiveness remain incompletely understood. **Methods**: This prospective cohort study included 182 men undergoing RP who completed a standardized physiotherapy program involving pelvic floor muscle exercises, biofeedback (BFB), and ultrasound-guided training. UI severity was assessed using the 1-h pad test and recorded absorbent product use. Outcomes were evaluated at three time points: one month, three months, and six months post-catheter removal. A multiple linear regression model was used to identify the predictors of continence improvement, defined as the change in pad test result between baseline and six months. **Results**: Pad test results and absorbent use decreased significantly across all UI severity stages (*p* < 0.0001). The greatest absolute improvement was observed in patients with severe incontinence at baseline (UI stage 3: mean reduction from 130.8 g to 23.7 g). Regression analysis revealed that only the baseline pad test result was significantly associated with the magnitude of improvement (β = 0.91; 95% CI: 0.85–0.97, *p* < 0.001; R^2^ = 0.89). Age, BMI, and time to rehabilitation were not significant predictors. **Conclusions**: Pelvic floor rehabilitation after RP is effective in improving continence, including in patients with severe baseline symptoms. The baseline pad test value emerged as the strongest predictor of rehabilitation response, highlighting the importance of initial assessment. These findings support the use of PFMT in clinical practice and emphasize the need for individualized treatment planning based on baseline UI severity.

## 1. Introduction

Radical prostatectomy (RP) is a standard surgical treatment for patients with localized prostate cancer [1]. Regardless of the operating modality, most patients experience functional deficits, with urinary incontinence (UI) being the most prominent [2]. This complication significantly decreases quality of life, and may raise concerns about uncontrolled urinary leakage, odor, and the need to use absorbent products such as pads and diapers. For many patients, it results in a decrease in physical, social, and intimate activity [3].

Estimates of post-RP UI vary widely, with the differences resulting mostly from divergent definitions and assessment methods. Depending on the source, the incidence ranges from 2.5% to as much as 90% [3,4,5]. Immediately after surgery, UI is observed in approximately 80% of patients; after 12 months the proportion decreases to 3–12% [5,6].

Pelvic floor muscle training (PFMT) plays an important role in the treatment of post-RP UI. Although evidence for PFMT efficacy remains mixed, both the European Association of Urology and the American Urological Association recommend it as first-line therapy [7,8,9,10,11,12,13]. The EAU advises educating patients about PFMT before surgery, and the AUA specifies that exercises should begin 3–4 weeks before RP [14,15].

Both guidelines also emphasize starting PFMT immediately after urethral catheter removal, when patients are highly motivated and often experience rapid functional gains, thereby expediting rehabilitation [16].

According to a systematic review by Chen et al. [17], timely initiation of PFMT results in a significantly faster return of urinary continence after RP. Motivation, however, tends to wane over time, particularly when early outcomes are suboptimal [16,18,19]. Accordingly, early physiotherapy is crucial for therapeutic success.

Post-RP UI rehabilitation is not confined to PFMT. Clinical practice also employs biofeedback (BFB), intra-anal or surface electrostimulation, magnetic stimulation, and manual therapy [8]. The pad test (PT) provides an objective measurement of UI severity. It compares the mass of special pads before and after one hour of activity, serving to stage UI, monitor therapeutic response, and predict continence recovery [20,21]. Another method of UI assessment is recording the number and types of absorbent products used.

Although numerous studies have assessed the effectiveness of PFMT, limited data identify which patient subgroups derive the greatest benefit. Most studies primarily examine the technical implementation of PFMT, while patient-related predictors of outcome remain underexplored.

Recent research increasingly emphasizes patient-specific factors—such as age, body mass index (BMI), disease stage, and key surgical parameters—in predicting the response to rehabilitation [22]. A better understanding of these dependencies enables more individualized and effective interventions, as well as motivating patients to actively participate in the rehabilitation process. Thus, robust and clinically applicable predictors of continence recovery are essential for designing personalized rehabilitation pathways following RP.

## 2. Materials and Methods

A total of 182 men scheduled for radical prostatectomy (RP) for prostate cancer were enrolled. Of these, 106 underwent laparoscopic RP (LRP) and 76 underwent robot-assisted RP (RARP). Both groups were included in a pooled analysis, as they underwent the same standardized rehabilitation protocol. Since the aim of this study was to evaluate the effectiveness of pelvic floor rehabilitation rather than surgical technique, no stratification by surgical approach was applied. This methodological decision is acknowledged as a limitation in the Section 4. The mean (SD) age was 66.1 (6.5) years. All patients were referred to a physiotherapist one month before surgery.

Inclusion criteria: adult men (≥18 years) undergoing radical prostatectomy for localized prostate cancer, with no prior neurological or urological disorders affecting continence, who provided written informed consent to participate in the study. Exclusion criteria: inability to attend physiotherapy due to medical contraindications, patient refusal, or incomplete clinical data.

### 2.1. Rehabilitation Protocol

The rehabilitation program consisted of 4 stages. At stage 0, one month before surgery, 146 patients took part in 3 physiotherapist-supervised meetings; 36 patients were unable to participate due to personal reasons. Pelvic floor muscle localization, activation, and control were taught utilizing surface electromyography (sEMG, Noraxon Ultium with an intra-anal probe and 50-mm surface electrodes [INTCO]), and Medison Sono Ace PICO ultrasound (US).

### 2.2. Detailed First Session Procedure

Patient instructions: teaching localization and voluntary activation of the pelvic floor muscles. Pelvic floor muscle examination: the patient is placed in the left lateral decubitus position, with the lower extremities flexed at the hip and knee joints; insertion of the intra-anal probe and placement of surface EMG electrodes is over the rectus abdominis and the right gluteal muscle, in accordance with SENIAM (Surface Electro Myo Graphy for the Non-Invasive Assessment of Muscles) project guidelines.

The sequence of pelvic floor exercises, according to the Glazer protocol [23], is as follows:

(1) Five fast contractions with immediate relaxation.
(2) Five 10-s contractions with 5-s interval.
(3) One 30-s contraction.
(4) Ejection of probe, electrode detachment.
(5) Evaluation and feedback.

During the two subsequent sessions, patients practiced controlled pelvic floor muscle contractions in the prone, sitting, and standing positions (isolated pelvic floor muscle contraction exercises; rapid contractions with immediate relaxation; pelvic floor muscle exercises coordinated with the breathing cycle). The patients were instructed to perform the pelvic floor muscle and breathing exercises four times a day, ten repetitions per set, in different body positions, with a particular focus on standing and sitting positions. Additionally, every patient received a set of lower extremity and pelvic girdle exercises to perform at home; they were also advised to walk for at least 30 min every day.

After the surgery and removal of the urinary catheter, the patients continued to perform the same exercises. Stage 1 rehabilitation commenced 1 month later. At the initial visit, a physiotherapist repeated the pelvic floor muscle examination and conducted a standardized 1-h pad test (Seni Man Level 4 Extra Plus) to quantify urinary leakage. The pad was weighed before placement beneath the penis inside the underwear, after which the participant completed the following activities: 15 min of drinking 0.5 L of water while seated; 30 min of walking along the corridor and on stairs; 10 sit-to-stand repetitions from a chair; 5 repetitions of lifting a weight from the floor; 1 min of jogging on the spot; 10 coughs; 1 min of hand-washing under running water.

The pad, sealed in a plastic bag at the end of the protocol, was then weighed again.WLC6/F1/R medical scale, with a readout accuracy of 0.1 g was used to weigh the pad. UI was defined as leakage of >2 g of urine.

Based on the PT readouts, the stage of UI was assessed as follows:

- Stage I: 2–10 g
- Stage II: 11–50 g
- Stage III: ≥50 g.

Number of used pads, as well as other utilized hygienic products (incontinence pads and adult diapers), was also recorded. During the 5 rehabilitation sessions, the patients performed controlled pelvic floor muscle and breathing exercises. Stages II and III of the physiotherapy program were delivered at 3 and 6 months after catheter removal. At each visit, the physiotherapist performed a control examination of pelvic floor muscles and PT. Participants who lost > 50 g of urine during the PT and/or displayed sensory deficits received adjunct neuromuscular electrostimulation, provided their serum prostate-specific antigen (PSA) level was within the normal range.

The following clinical variables, confirmed as predictive factors for urinary incontinence in various studies, were analyzed: age, BMI, UI stage, interval from surgery to initiation of rehabilitation, and PT result [16,24,25,26]. There were no losses to follow-up; all 182 patients completed the full rehabilitation protocol and were assessed at all 3 postoperative time points.

This study received approval by the Bioethics Committee of Collegium Medicum, Jan Kochanowski University, Kielce, Poland (approval no. 34/2018, approval date: 11 June 2018).

### 2.3. Statistical Analyses

Statistical analyses were performed using R software (version 4.4.1). Continuous variables were expressed as mean (standard deviation, SD) or median (interquartile range, IQR), depending on the distribution of the variable. Categorical variables are summarized as counts and percentages. Differences in absorbent use (pads, diapers, and total absorbents) over time were assessed using the Friedman test for repeated measures within each baseline UI stage group. When significant, post hoc pairwise comparisons were conducted with Bonferroni correction for multiple testing. Differences between baseline UI stage groups at each time point were assessed using the Kruskal–Wallis test. If statistically significant, pairwise Mann–Whitney U tests with Bonferroni adjustment were used as post hoc analyses. Changes in categorical variables (e.g., number of pads/diapers used per day: 0, 1, 2, 3+) were analyzed using the Chi-square test of independence. To assess the predictors of continence improvement, a multiple linear regression model was constructed using the change in pad test result between Examination 1 and Examination 3 (Δ Pad test, in grams) as the dependent variable. Assumptions of linearity, normality of residuals, and homoscedasticity were verified prior to model interpretation. The baseline pad test value was included in the model to account for ceiling effects. Candidate predictors were selected based on clinical relevance, previous literature, and data completeness, and included UI stage at baseline, age, BMI, time from surgery to rehabilitation, and baseline pad test result. Results are presented as unstandardized regression coefficients (β) with 95% confidence intervals and corresponding *p*-values. A two-tailed *p*-value of <0.05 was considered statistically significant, unless otherwise specified.

## 3. Results

A total of 182 patients who underwent radical prostatectomy were included in the analysis. The mean age of participants was 66.1 years (SD 6.5), and the mean BMI was 28.2 kg/m^2^ (SD 3.6). Most patients (80.2%) participated in preoperative pelvic floor rehabilitation. The median time from surgery to initiation of rehabilitation was 36.1 days (SD 14.0). At baseline, 35.2% of patients had no urinary incontinence (UI stage 0), while 26.4% were classified as UI stage 3. The mean pad test result at baseline was 43.9 g (SD 68.9), which decreased to 8.0 g (SD 22.9) after rehabilitation. The mean improvement (Δ Pad test) between Examinations 1 and 3 was 36.0 g (SD 63.4), indicating a substantial recovery in urinary continence across the cohort. All 182 patients completed the rehabilitation protocol and follow-up assessments. Additional clinical and oncological characteristics are summarized in Table 1.

**Figure 1:**
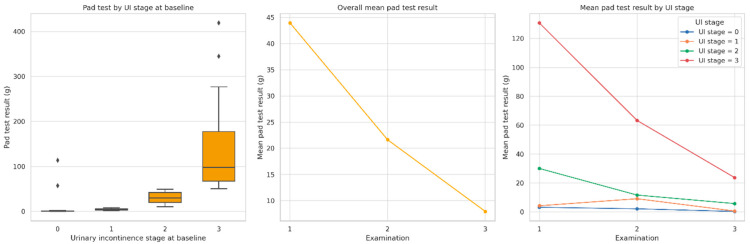
Distribution and changes in pad test results (g) across three postoperative examinations. Note: The left panel presents baseline pad test values by urinary incontinence (UI) stage. The middle panel shows overall mean improvement over time. The right panel illustrates mean changes stratified by UI stage. All comparisons were statistically significant (*p* < 0.0001, Bonferroni corrected). Statistical comparisons were performed using the Friedman test for repeated measures.

Pad test results significantly improved across the entire cohort over the course of rehabilitation. As shown in Figure 1, the mean amount of urine loss decreased consistently between Examination 1 and Examination 3, both overall and within each UI stage at baseline. Table 2 presents detailed mean values and statistical comparisons for each UI group. Significant reductions in pad test results were observed over time in all groups (*p* < 0.0001, Friedman test). The greatest absolute improvement was seen in patients with baseline UI stage 3 (from 130.8 g to 23.7 g), while those with no incontinence at baseline showed minimal change. Post hoc comparisons revealed that differences between UI stage groups were statistically significant at all examinations, with some overlap at Examination 3 (see notes a–d in Table 2).

A significant reduction in absorbent use was observed over time, both in terms of pads and diapers. As shown in Table 3 and Table 4, the proportion of patients not using any pads increased steadily from Examination 1 to Examination 3 across all UI stage groups. At baseline, only 36% of UI stage 0 patients and 6% of UI stage 3 patients reported zero pad use; by Examination 3, these proportions rose to 72% and 42%, respectively. A similar trend was observed in diaper use. Among patients with UI stage 3, the proportion not using any diapers increased from just 8% at Examination 1 to 77% by Examination 3. All within-stage changes were statistically significant (*p* < 0.0001 for all pad comparisons; *p* = 0.0179 for final diaper comparison in Table 4; Chi-square test of independence).

**Figure 2:**
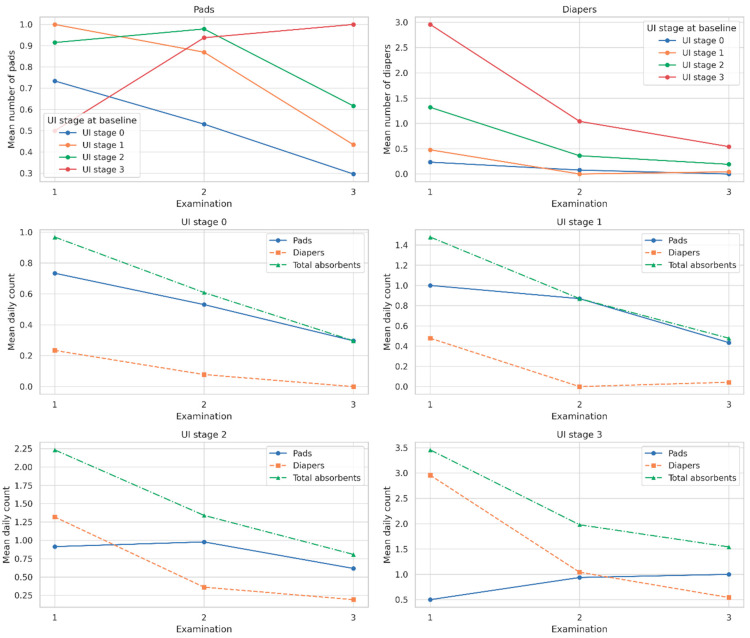
Mean number of pads, diapers, and total absorbents used per day across three postoperative examinations, shown separately by urinary incontinence (UI) stage at baseline (stages 0–3). Note: Solid lines represent pads, dashed lines represent diapers, and dash-dot lines indicate total absorbents (pads + diapers). A consistent reduction in absorbent use over time was observed in all UI stage groups. Statistical significance of within-group changes was confirmed using the Friedman test (*p* < 0.0001 for all comparisons, Bonferroni corrected).

As shown in Table 5 and Figure 2. among patients with UI stage 3, total absorbent use declined from 3.5 to 1.5 per day; similar downward trends were observed in UI stages 2, 1, and 0. The number of diapers used decreased markedly in higher UI stages. For example, mean diaper use in stage 3 dropped from 3.0 at Examination 1 to 0.5 at Examination 3, while pad use slightly increased from 0.5 to 1.0, possibly indicating a transition to lighter absorbents. All within-group reductions in total absorbent mean absorbent use decreased consistently across all baseline UI stages over time; use was statistically significant (*p* < 0.0001, Friedman test). Moreover, significant differences in absorbent use between UI stage groups were observed at each examination point (*p* < 0.0001, Kruskal–Wallis test).

**Figure 3:**
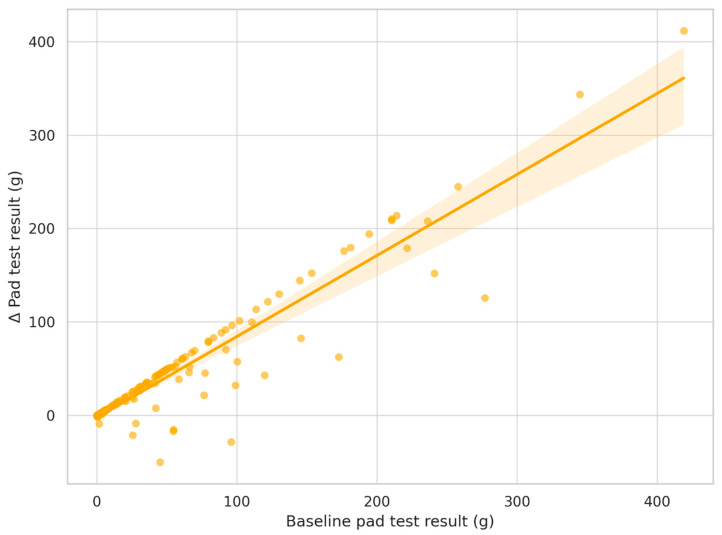
Relationship between baseline incontinence severity and improvement in pad test result. Note: Scatterplot shows the association between baseline pad test result (g) and the change (Δ) in pad test result between Examination 1 and Examination 3. The regression line and 95% confidence interval are shown. Each dot represents an individual patient. Regression line with 95% confidence interval shaded. Greater initial incontinence was associated with greater improvement (*p*< 0.001, *R*^2^ = 0.89).

Results of the multiple linear regression analysis examining predictors of improvement in urinary continence, defined as the difference in pad test result (grams) between Examination 1 and Examination 3, are presented in Table 6 and Figure 3. The included predictors were selected based on clinical relevance and data availability as follows: baseline UI stage, age, BMI, time from surgery to rehabilitation, and baseline pad test result. The final model explained 89% of the variance in pad test improvement (*R*^2^ = 0.894), indicating excellent model fit. Only the baseline pad test result was significantly associated with improvement (*p* < 0.001), indicating that patients with more severe incontinence at baseline experienced greater absolute improvement. The baseline UI stage was marginally non-significant (*p* = 0.059), while age, BMI, and time to rehabilitation were not statistically significant predictors.

## 4. Discussion

Our study shows that post-RP rehabilitation significantly improves urinary continence, as evidenced by a reduction in the PT score and decreased use of hygienic products. The mean pad test result score decreased significantly after the implementation of the therapeutic program. The largest improvement was observed between the first and the third assessments, indicating progressive recovery of pelvic floor muscle function. A progressively larger proportion of patients discontinued pad use, paralleling gains in quality of life and comfort in daily activities. These findings are consistent with previous reports supporting the efficacy of personalized physiotherapy in the treatment of UI [7,14,27]. According to previous studies, specialist-supervised interventions (including patient instruction, US, and BFB) may be more effective than unsupervised exercise-based programs, although both approaches yield significant benefits [7,16,28].

Many patients struggle to identify and contract their pelvic floor muscles correctly. Therefore, specialist-supervised therapy utilizing US and BFB is highly recommended, particularly in patients struggling to correctly engage pelvic floor muscles. US enables real-time pelvic floor muscle visualization, whereas BFB supplies immediate feedback on exercise quality. Baumann et al. likewise reported superior outcomes when training was conducted under controlled conditions [14]. Although the effectiveness of PFMT has been confirmed in multiple publications, some evidence questions whether it is superior to other rehabilitation strategies [7]. Taken together, these findings highlight the importance of tailoring rehabilitation protocols to individual patient needs and capabilities.

These discrepancies also apply to the use of BFB and electrostimulation; nevertheless, a 2023 meta-analysis demonstrated that each modality leads to an improvement in pelvic floor muscle function at different stages of rehabilitation [16].

UI significantly diminishes quality of life, precipitating stress, anxiety, and a decrease in self-esteem. The physiotherapist plays a pivotal role not only in administering treatment, but also in educating patients—including in the use of techniques that reduce the risk of leakage in daily activities (e.g., coughing into a flexed elbow). 

Preoperative physiotherapy interventions did not result in a statistically significant improvement in continence outcomes in our cohort. A 2023 meta-analysis reported comparable results, finding no clear benefit of preoperative exercise on post-RP urinary continence [10]. Conversely, other authors have suggested that introducing physiotherapy approximately two months before RP may enhance continence recovery [29].

Divergent findings may reflect variations in exercise type, repetition volume, and execution quality. Evidence indicates that early, correctly performed training can augment neuroplasticity, and promote post-surgical muscle adaptation [22]. Preoperative programs also confer practical and educational advantages. Patients learn to activate pelvic floor muscles in daily life, which leads to better awareness of one’s body, and better initiation of post-RP exercises. Although the preoperative regimen in our group did not reach statistical significance, it may nevertheless offer clinical benefits, especially in terms of patient education, confidence, and early self-management. It should be noted, however, that 36 patients were unable to attend the preoperative sessions. Although they did not differ significantly in baseline incontinence severity and participated in the postoperative program, their lower preoperative engagement may have introduced a degree of bias and influenced the overall outcomes.

### 4.1. Physiotherapy Effectiveness by Urinary Incontinence Severity

The greatest post-rehabilitation gains were observed in patients with stage III UI. In stage 0 (urinary leakage lower than 2 g) the difference was negligible, suggesting that rehabilitation may be most beneficial in patients with more severe incontinence (i.e., >50 g of urine loss in the pad test one month after RP). High baseline PT scores were predictive of higher improvement potential [21,30]. However, this strong association may partially reflect regression to the mean, particularly in patients with extreme initial incontinence values.

Repeated PT assessments also conferred motivational benefits. For participants who scored better, the test functioned as a reward, whereas in the absence of progress, it prompted renewed engagement with the program. Furthermore, for some patients, the test served as a justification for discontinuing protective pads, which—although they offer a sense of security—may also inadvertently foster acceptance of partial continence and limit further improvement.

In our subgroup analysis (Table 6), one of the associations approached but did not reach conventional statistical significance (*p* = 0.059). This finding should therefore be interpreted as a trend rather than a definitive effect. While not statistically significant, it may still reflect a clinically relevant pattern that warrants further investigation in larger samples.

### 4.2. Demographic and Clinical Factors

Age, BMI, and the interval between surgery and the start of physiotherapy did not significantly affect rehabilitation outcomes. These findings differ from several earlier studies, which identify BMI and advanced age as risk factors for the development of post-RP UI [31,32]. One possible explanation is that participation in a structured physiotherapy program may have mitigated the influence of these demographic factors. Supervised and personalized rehabilitation might reduce variability attributable to age or BMI by standardizing motor learning and functional pelvic floor activation across patients. Further studies are needed to explore this hypothesis in stratified populations. Other variables, such as prostate volume and intra-operative factors, may exert a greater influence on prognosis [33,34]. Future studies should consider incorporating surgical details, such as nerve-sparing techniques or intra-operative complications.

One limitation of this study is the pooling of patients undergoing laparoscopic and robot-assisted prostatectomy. Although both groups followed an identical rehabilitation protocol, differences in surgical technique could theoretically influence continence recovery. However, since the primary objective was to assess the effectiveness of rehabilitation, not surgical outcomes, we considered this approach appropriate and methodologically justified. Our results suggest that not every patient should necessarily follow a uniform post-RP rehabilitation protocol. Tailoring therapy based on UI severity and clinical context is essential for optimal outcomes.

## 5. Conclusions

Pelvic floor rehabilitation following radical prostatectomy is effective in significantly improving urinary continence, as demonstrated by marked reductions in pad test results and absorbent product use. The greatest improvement was observed among patients with more severe baseline incontinence, indicating that even those with advanced symptoms can benefit from rehabilitation. Among the clinical variables analyzed, only the initial pad test result was significantly associated with the magnitude of improvement, underscoring the importance of baseline severity as a predictor of therapeutic response. Age, body mass index, and time from surgery to rehabilitation initiation were not significantly associated with outcomes, suggesting that pelvic floor rehabilitation may be broadly effective across diverse clinical subgroups. These findings support the continued integration of pelvic floor rehabilitation into standard post-prostatectomy care and emphasize the value of baseline continence severity in guiding patient counseling and individualized treatment planning.

## Figures and Tables

**Table 1:** Baseline characteristics of the study group.

Characteristic	Total Group (n = 182)
Age (years), mean (SD)	66.1 (6.5)
BMI (kg/m^2^), mean (SD)	28.2 (3.6)
Preoperative PSA (ng/mL), mean (SD)	9.2 (7.8)
Postoperative PSA (ng/mL), mean (SD)	0.2 (1.5)
Urinary incontinence stage (at baseline), n (%)	
Stage 0	64 (35.2)
Stage 1	23 (12.6)
Stage 2	47 (25.8)
Stage 3	48 (26.4)
Rehabilitated before surgery, n (%)	
No	36 (19.8)
Yes	146 (80.2)
Time to rehabilitation (days), mean (SD)	36.1 (14.0)
Pad test result at Examination 1(g), mean (SD)	43.9 (68.9)
Pad test result at Examination 3 (g), mean (SD)	8.0 (22.9)
Δ Pad test result (g), mean (SD)	36.0 (63.4)
Type of surgery, n (%)	
LRP	106 (58.2)
RARP	76 (41.8)
pT, n (%)	
pT1	2 (1.1)
pT2	126 (69.2)
pT3	54 (29.7)
pN, n (%)	
Nx	17 (9.3)
N0	163 (89.6)
N1	2 (1.1)
pM, n (%)	
M0	14 (7.7)
Mx	168 (92.3)
GS1, n (%)	
GS1 → 3	58 (31.9)
GS1 → 4	121 (66.5)
GS1 → 5	3 (1.6)
GS2, n (%)	
GS2 → 3	77 (42.3)
GS2 → 4	89 (48.9)
GS2 → 5	16 (8.8)
GS, n (%)	
GS → 6	16 (8.8)
GS → 7	100 (54.9)
GS → 8	50 (27.5)
GS → 9	16 (8.8)
Persistent PSA, n (%)	
No	158 (86.8)
Yes	24 (13.2)
EPE, n (%)	
EPE 0	129 (70.9)
EPE 1	47 (25.8)
EPE 2	6 (3.3)
SVI, n (%)	
No	162 (89.0)
Yes	20 (11.0)
EAU, n (%)	
EAU 1	9 (4.9)
EAU 2	139 (76.4)
EAU 3	34 (18.7)
ISUP, n (%)	
ISUP 1	16 (8.8)
ISUP 2	39 (21.4)
ISUP 3	61 (33.5)
ISUP 4	50 (27.5)
ISUP 5	16 (8.8)

Abbreviations: BMI, body mass index; PSA, prostate-specific antigen; LRP, laparoscopic radical prostatectomy; RARP, robot-assisted radical prostatectomy; pT, pathological tumor stage; pN, pathological nodal stage; pM, pathological metastatic stage; GS1, primary Gleason pattern; GS2, secondary Gleason pattern; GS, total Gleason score (GS1 + GS2); EPE, extra-capsular extension; SVI, seminal vesicle invasion; EAU, European Association of Urology, ISUP, International Society of Urological Pathology; SD, standard deviation. Note: Values are presented as mean (standard deviation) unless otherwise indicated. Pad test result reflects urinary loss in grams during a standardized 1-h pad test. Δ Pad test result indicates the difference between Examinations 1 and 3, with higher values representing greater improvement. EAU risk groups: EAU1 = low risk (PSA < 10 ng/mL, ISUP ≤ 1, cT1–T2a); EAU2 = intermediate risk (PSA 10–20 ng/mL, ISUP 2–3, cT2b–T2c); EAU3 = high risk (PSA > 20 ng/mL, ISUP ≥ 4, ≥T3). ISUP Grade Groups correspond to Gleason-based grading: ISUP 1 = Gleason ≤ 6, ISUP 2 = 3 + 4, ISUP 3 = 4 + 3, ISUP 4 = 8, ISUP 5 = 9–10. GS1 = primary Gleason pattern; GS2 = secondary Gleason pattern; GS = combined Gleason score (GS1 + GS2).

**Table 2:** Changes in pad test results over time by urinary incontinence stage. Mean (SD), *p*-values from Friedman test.

UI Stageat Baseline	Examination 1	Examination 2	Examination 3	*p*
Stage 0	3.2 (15.7) ^a^	2.2 (14.2) ^b^	0.4 (1.4) ^c^	0.0001
Stage 1	4.3 (1.7) ^a^	9.1 (36.2) ^b^	0.6 (0.7) ^d^	0.0001
Stage 2	30.1 (12.4) ^a^	11.7 (15.2) ^b^	5.8 (16.4) ^c^	0.0001
Stage 3	130.8 (83.1) ^a^	63.3 (72.0) ^b^	23.7 (37.3) ^c,d^	0.0001

Post hoc tests with Bonferroni corrections; ^a^ In Examination 1, significant differences were observed between all UI stage groups; ^b^ In Examination 2, significant differences were observed between all UI stage groups; ^c^ In Examination 3, significant differences were observed between stage 0 and stage 2, and between stage 0 and stage 3; ^d^ In Examination 3, a significant difference was also found between stage 1 and stage 3.

**Table 3:** Distribution of daily pad use across UI stage groups at each examination point with Chi-square tests of independence.

Number of Pads	Examination	UI Stage 0	UI Stage 1	UI Stage 2	UI Stage 3	*p* (Chi^2^)
0, n (%)	Examination 1	23 (35.9)	6 (26.1)	29 (61.7)	38 (79.2)	<0.0001
	Examination 2	30 (46.9)	5 (21.7)	19 (40.4)	24 (50.0)	<0.0001
	Examination 3	46 (71.9)	13 (56.5)	20 (42.6)	20 (41.7)	<0.0001
1, n (%)	Examination 1	36 (56.2)	12 (52.2)	5 (10.6)	3 (6.2)	<0.0001
	Examination 2	34 (53.1)	16 (69.6)	15 (31.9)	12 (25.0)	<0.0001
	Examination 3	17 (26.6)	10 (43.5)	25 (53.2)	16 (33.3)	<0.0001
2, n (%)	Examination 1	4 (6.2)	4 (17.4)	6 (12.8)	3 (6.2)	<0.0001
	Examination 2	0 (0.0)	2 (8.7)	9 (19.1)	5 (10.4)	<0.0001
	Examination 3	1 (1.6)	0 (0.0)	2 (4.3)	6 (12.5)	<0.0001
≥3, n (%)	Examination 1	1 (1.6)	1 (4.3)	7 (14.9)	4 (8.3)	<0.0001
	Examination 2	0 (0.0)	0 (0.0)	4 (8.5)	7 (14.6)	<0.0001
	Examination 3	0 (0.0)	0 (0.0)	0 (0.0)	6 (12.5)	<0.0001

Note: UI stage groups compared separately at each examination time point using Chi-square tests.

**Table 4:** Distribution of daily diaper use across UI stage groups at each examination point with Chi-square tests of independence.

Number of Diapers	Examination	UI Stage 0	UI Stage 1	UI Stage 2	UI Stage 3	*p* (Chi^2^)
0, n (%)	Examination 1	56 (87.5)	16 (69.6)	16 (34.0)	4 (8.3)	<0.0001
	Examination 2	62 (96.9)	23 (100.0)	36 (76.6)	27 (56.2)	<0.0001
	Examination 3	64 (100.0)	22 (95.7)	42 (89.4)	37 (77.1)	0.0179
1, n (%)	Examination 1	3 (4.7)	3 (13.0)	14 (29.8)	3 (6.2)	<0.0001
	Examination 2	0 (0.0)	0 (0.0)	6 (12.8)	2 (4.2)	<0.0001
	Examination 3	0 (0.0)	1 (4.3)	3 (6.4)	4 (8.3)	0.0179
2, n (%)	Examination 1	3 (4.7)	4 (17.4)	9 (19.1)	9 (18.8)	<0.0001
	Examination 2	1 (1.6)	0 (0.0)	4 (8.5)	12 (25.0)	<0.0001
	Examination 3	0 (0.0)	0 (0.0)	1 (2.1)	4 (8.3)	0.0179
≥3, n (%)	Examination 1	2 (3.1)	0 (0.0)	8 (17.0)	32 (66.7)	<0.0001
	Examination 2	1 (1.6)	0 (0.0)	1 (2.1)	7 (14.6)	<0.0001
	Examination 3	0 (0.0)	0 (0.0)	1 (2.1)	3 (6.2)	0.0179

**Table 5:** Mean number of pads, diapers, and total absorbents by baseline UI stage across the three postoperative examinations.

UI Stage at Baseline	Examination	Mean Number of Pads	Mean Number of Diapers	Mean Number of Total Absorbents
Stage 0	1	0.7	0.2	1.0
	2	0.5	0.1	0.6
	3	0.3	0.0	0.3
Stage 1	1	1.0	0.5	1.5
	2	0.9	0.0	0.9
	3	0.4	0.0	0.5
Stage 2	1	0.9	1.3	2.2
	2	1.0	0.4	1.3
	3	0.6	0.2	0.8
Stage 3	1	0.5	3.0	3.5
	2	0.9	1.0	2.0
	3	1.0	0.5	1.5

**Table 6:** Predictors of improvement in the pad test result (Δ Pad test).

Characteristic	β	CI 2.5%	CI 97.5%	*p*
Intercept	28.622	−15.214	72.459	0.199
UI stage at baseline	−3.327	−6.78	0.126	0.059
Age	−0.306	−0.794	0.182	0.218
BMI	−0.174	−1.03	0.683	0.69
Time to rehabilitation	−0.075	−0.295	0.145	0.503
Baseline pad test result	0.909	0.849	0.97	<0.001

Abbreviations: UI, urinary incontinence; BMI, body mass index; CI, confidence interval. Note: Linear regression model predicting change in pad test result (Δ Pad test result = Examination 1 − Examination 3). CI = confidence interval. UI = urinary incontinence.

## Data Availability

The data supporting the findings of this study are not publicly available due to privacy and ethical restrictions. Reasonable requests for access to anonymized data may be considered by the corresponding author.
